# Supplementary material for: Evidence supported by Mendelian randomization: impact on inflammatory factors in knee osteoarthritis
Source: Front Med (Lausanne). 2024 May 28;11:1382836. doi: 10.3389/fmed.2024.1382836 (PMC11165061; doi:10.3389/fmed.2024.1382836)
Supplement: Supplementary file 1 [file Data_Sheet_1.ZIP › data/GCST90274843_5e6/843.leaveoneout.pdf]

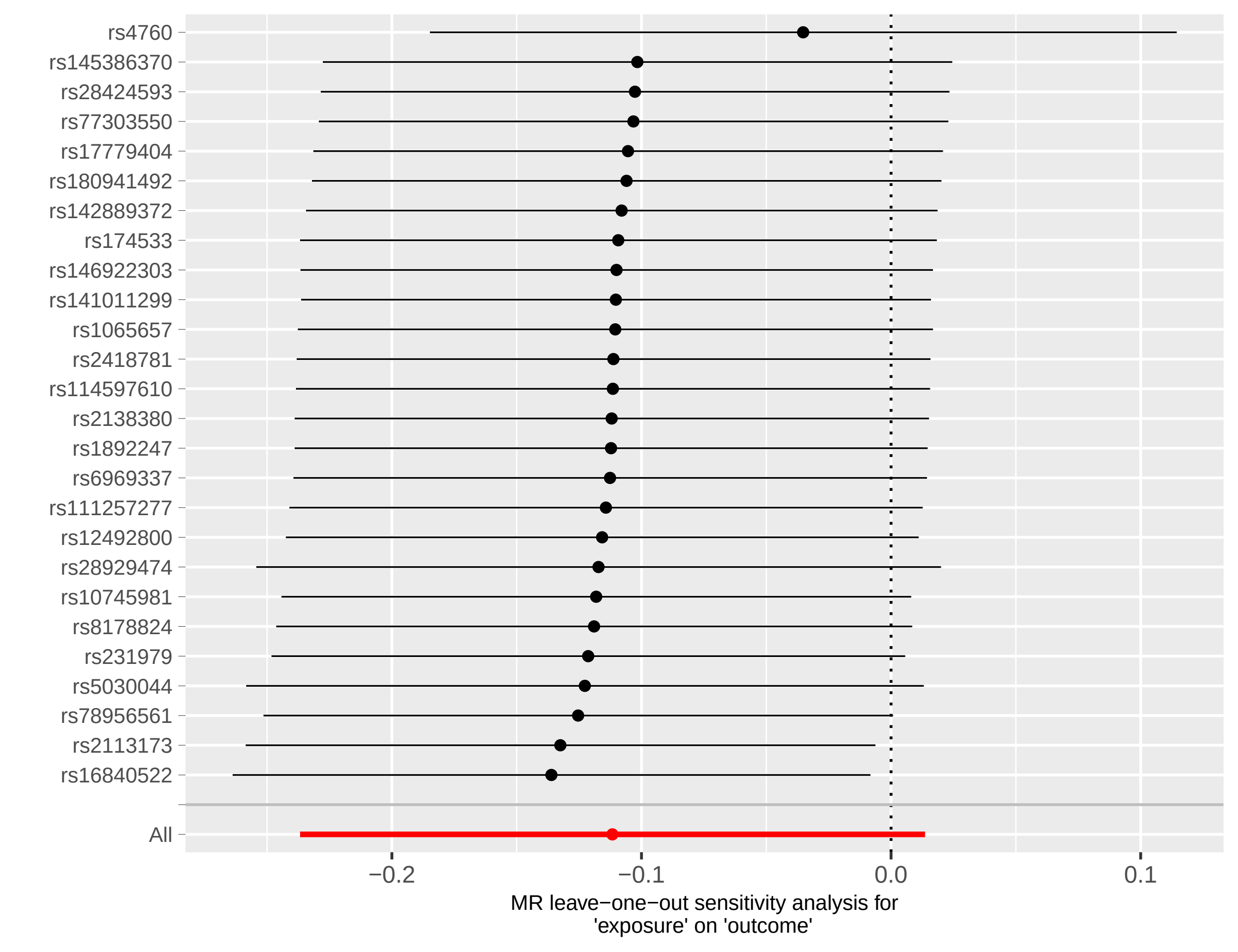
rs4760
rs145386370
rs28424593
rs77303550
rs17779404
rs180941492
rs142889372
rs174533
rs146922303
rs141011299
rs1065657
rs2418781
rs114597610
rs2138380
rs1892247
rs6969337
rs111257277
rs12492800
rs28929474
rs10745981
rs8178824
rs231979
rs5030044
rs78956561
rs2113173
rs16840522
All
−0.2
−0.1
0.0
0.1
MR leave−one−out sensitivity analysis for
'exposure' on 'outcome'
